# Viral Phylogenomics Using an Alignment-Free Method: A Three-Step Approach to Determine Optimal Length of *k-mer*

**DOI:** 10.1038/srep40712

**Published:** 2017-01-19

**Authors:** Qian Zhang, Se-Ran Jun, Michael Leuze, David Ussery, Intawat Nookaew

**Affiliations:** 1 UT-ORNL Graduate School of Genome Science and Technology, University of Tennessee, Knoxville, TN 37996, USA; 2 Comparative Genomics Group, Biosciences Division, Oak Ridge National Laboratory Oak Ridge, TN 37831 USA; 3 Department of Biomedical Informatics, College of Medicine, University of Arkansas for Medical Sciences, Little Rock, AR 72205, USA; 4 Joint Institute for Computational Sciences, University of Tennessee, Knoxville, TN 37831, USA; 5 Computational Biomolecular Modeling and Bioinformatics Group, Computer Science and Mathematics Division, Oak Ridge National Laboratories, Oak Ridge, TN 37831, USA

## Abstract

The development of rapid, economical genome sequencing has shed new light on the classification of viruses. As of October 2016, the National Center for Biotechnology Information (NCBI) database contained >2 million viral genome sequences and a reference set of ~4000 viral genome sequences that cover a wide range of known viral families. Whole-genome sequences can be used to improve viral classification and provide insight into the viral “tree of life”. However, due to the lack of evolutionary conservation amongst diverse viruses, it is not feasible to build a viral tree of life using traditional phylogenetic methods based on conserved proteins. In this study, we used an alignment-free method that uses *k*-mers as genomic features for a large-scale comparison of complete viral genomes available in RefSeq. To determine the optimal feature length, *k* (an essential step in constructing a meaningful dendrogram), we designed a comprehensive strategy that combines three approaches: (1) cumulative relative entropy, (2) average number of common features among genomes, and (3) the Shannon diversity index. This strategy was used to determine *k* for all 3,905 complete viral genomes in RefSeq. The resulting dendrogram shows consistency with the viral taxonomy of the ICTV and the Baltimore classification of viruses.

Whole-genome sequencing of pathogens is now commonly used^1^,^2^,^3^, and was made possible by exponential reductions in the cost of sequencing^4^ and computational advances in biological sequence analysis^5^,^6^. Viral taxonomy, in particular, has benefited from the availability of many new viral-genome sequences, enabling the improved classification of viruses. In support of viral genomics research, the NCBI Viral Genome Project^7^ provides thousands of viral reference sequences, which cover a wide range of viral taxonomic families in the NCBI Reference Sequence Database. The classification of viruses is maintained by the International Committee on Taxonomy of Viruses (ICTV), which considers multiple viral properties and consensus data^8^, including similarities in genome structures, host ranges, and the presence of homologous genes and various phylogenetic features^9^. Although viral taxa have been continuously updated by the virus research community^10^,^11^, there are still many misclassifications in the ICTV viral taxonomy^12^, as well as many viral families currently not included in the RefSeq database. Further, the sequencing of viral metagenomic samples often results in many viral genomes that are of unknown origin^13^,^14^.

Phylogenetic analysis is widely used for taxonomic classification, characterization, and revision^15^,^16^. However, for prokaryotic genomes, phylogenetic trees based on small subunit rRNAs often do not agree with those based on different genes. Conflicts among gene trees have increased as more genes and genomes are sequenced^17^. This incongruence can have many causes, including tree-building errors, incomplete lineage sorting, hidden paralogy, and horizontal gene transfer. As early as 1996, inconsistent phylogenetic trees were obtained for viruses when using different numbers of isolates, or when different lengths of aligned sequences were used (as in a study of hepatitis C viruses^18^). Similar inconsistencies have been reported for human papillomaviruses^19^, SARS coronavirus^20^, and some plant viruses^21^.

Phylogenomic dendograms constructed using whole-genome sequences are based on a more complete set of genomic information than phylogenies based on individual genes^22^. For large-scale comparisons of genome-scale sequences, especially highly diverse ones, alignment-free methods of phylogeny construction have been increasingly used in the past few years^23^,^24^,^25^,^26^. There are two categories of alignment-free methods for phylogenomic analysis: one based on the statistics of word frequency, the other on Kolmogorov complexity and chaos theory^27^. The primary advantage of these methods is that they enable quick genome-scale comparisons with linear time complexity (O(n))^28^ more efficiently than minimum likelihood or Bayesian alignment methods with subquadratic time complexity (o(n^2^)). Another advantage of alignment-free methods is that they can be used to compare sequences from draft genomes, with information loss proportional to the number of discontinuities in a genome. However, alignment-free methods do not capture the nuances of evolutionary models that incorporate site-dependent substitution patterns. Therefore, it is not possible to interpret branch lengths of alignment-free based trees in terms of mutation rates, even though alignment-free trees constructed from whole-genome sequences capture taxonomic classification (which reflects the evolutionary history of organisms) better than 16 S rRNA alignment-based trees for prokaryotes^28^.

Sims *et al*.^29^ introduced an alignment-free method that uses a measure based on the Jensen–Shannon divergence between feature frequency profiles (FFPs), where the features, called *k*-mers, are short nucleotide or amino-acid sequences of length *k*. Applied in eukaryotic and prokaryotic systems, this approach shows great consistency with the taxonomic information accepted by the scientific community^30^,^31^. For viruses, Wu *et al*.^32^ applied the FFP method to whole-proteome sequences of 142 large dsDNA eukaryotic viruses, and Huang *et al*. used this approach when evaluating different methods for phylogenetic analysis of multiple-segmented viruses^33^,^34^. To date, however, relatively little work has been done using FFP to determine the phylogeny of virus genomes^35^, and there are only a few reports^36^,^37^ on the construction of phylogenetic trees from the thousands of known viral genomes.

In general, genome-scale phylogenetic trees can be built using either whole-genome sequences or whole-proteome sequences. However, some viruses have only one or two genes from which protein sequences can be predicted, and viral proteins tend to be very diverse. As a consequence, it is not feasible to build a viral “tree of life” based on conserved proteins. Therefore, we have used an FFP approach applied to complete viral genome sequences, and have built a dendogram of viruses.

A major challenge in using the FFP method for comparing whole genomes is determining the optimal *k*-mer length. In previous studies of dsDNA eukaryotic viruses^15^,^16^,^21^, the optimal feature length was based on cumulative relative entropy (CRE) and relative sequence divergence (RSD). For each individual genome and a value of *k*, the CRE (determined by a comparison of the observed FFP and the expected FFP from a second-order Markov model) captures how much information from the whole-genome sequence is encoded in the FFP. In other words, CRE indicates the power of the FFP to reconstruct the whole-genome sequence. Smaller CRE values, which result from longer *k*-mers, are indicative of the ability to better identify individual genomes. For a whole genome, the RSD for a value of *k* is a measure of the relatedness of the genome sequence (in terms of FFP) to a random sequence of the same length. According to Wu *et al*.^32^, the optimal value of *k* is the value when both CRE and RSD decrease to <10% of their maximum values as *k* is increased.

Determining RSD values becomes increasingly computationally complex as the number of genomes grows. This increase in complexity is due, in part, to an increase in the density of the *k*-mer feature space. We found that RSD cannot monotonically decrease when *k* increases, which is probably because this huge dimensional *k*-mer space can cover artificial *k*-mers (*k*-mers derived from random sequences), even though their probabilities are quite low.

In this study, we consider the 3,905 complete viral genomes that are available in the NCBI Reference Sequence Database (RefSeq)^38^. We show that CRE is significantly influenced by the genome size, as well as *k*-mer composition. Genomes of different sizes show different trend CRE curves. For small viral genomes (~3 kb), CRE values drop to zero around a *k* value of 6; for large viral genomes (1 Mb or more), the drop increases to a *k* value of 10. Consequently, CRE values for genomes of greatly varied sizes cannot simultaneously be decreased to <10% of maximum values at the fixed-feature length as suggested by Wu *et al*.^32^. Therefore, we first grouped viral genomes by genomic size. For each group, we proposed the optimal *k*-mer length by considering several genomic features, including the CRE value, the number of *k*-mers shared by genomes, and the total number of *k*-mers observed, and then we constructed a dendrogram at its optimal *k*-mer length. Finally, we derived a procedure to decide the optimal feature length to compare all 3,905 complete viral genomes. The tree of life for viral whole genomes constructed by our alignment-free method can be visualized using the optimal feature length for the global view.

## Results

### Dataset and information content evaluation

**Figure 1:**
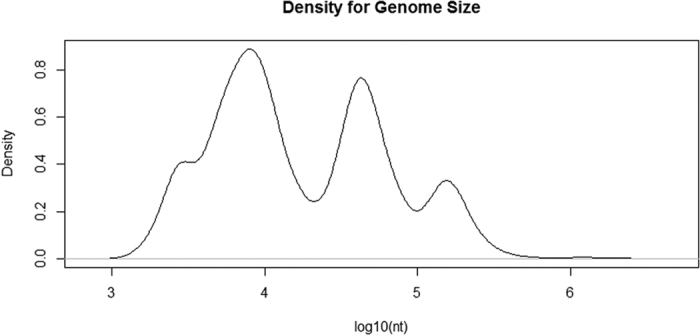
Distribution of genome size for 3,905 viral genomes in a semi-logX scale.

**Figure 2:**
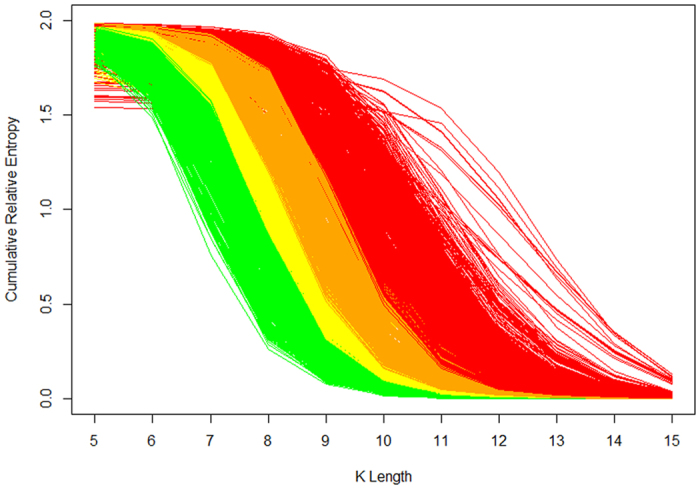
CRE curves for the 3,905 viral RefSeq genomes. The curves start to fall to <10% of the maximum at *k* = 9 and most genomes satisify the criteria at *k* = 13. Subgroups Q1, Q2, Q3, and Q4 are green, yellow, orange, and red, respectively.

The non-redundant dataset includes 3,905 complete genomes from the RefSeq viruses as summarized in Supplementary Table S1. The smallest genome is the *Anguilla anguilla* circovirus (NC_023421), with a length of 1,378 nt and the largest genome is *Pandoravirus salinus* (NC_022098), which is 2,473,870 nt long. The distribution of genome sizes is depicted in the density plot in Fig. 1. The long tail on the right shows some large genome sizes as outliers, such as *Pandoraviruses, Megaviruses, Mimiviruses,* and other giant viruses. It is worth mentioning that, after determining the CRE values as shown in Fig. 2, we noted that the recommended range for *k*-mer length varies greatly depending on genome size, and therefore divided the dataset into four arbitrary subgroups (Q1–Q4) using the 25%, 50%, and 75% quartiles cut-off of 6,407; 12,141; and 45,242 bp; respectively.

### Assessment of the optimal feature length (*k*)

Because the criteria used by Wu *et al*.^32^ were not directly applicable to our large-scale virus dataset, due to the dependence of CRE on genome size, we determined the optimal feature length based on three criteria: (1) from an individual genome perspective, using CRE to find the minimum feature length: where the genome curves reach zero CRE or fall to <10% of their CRE maximum values; this CRE value was the original criterion for optimal feature lengths in previously published papers^31^,^32^,^39^; (2) from a pairwise-comparison perspective, the average number of common features (ACF) among genomes was applied to determine the maximum feature length: the length prior to ACF dropping to a lower value; this ACF criterion is defined as the average number of common features when comparing pairwise to each of the other genomes at a specific feature length; (3) from an “all-genomes comparison” perspective, we measured commonness of *k*-mers among all genomes in our dataset in terms of the diversity index to narrow down the range of optimal feature length. The Shannon diversity index was used to quantify the diversity of commonness of *k*-mers using the fraction of *k*-mers shared by genomes. The preferred length is the one with a higher Shannon diversity index value (which represents more diversity in the commonness of *k*-mers) in the range suggested from criteria 1 and 2. When the three steps suggest multiple optimal lengths in the range, then the tree stability (based on Robinson–Foulds distance) is also considered supporting information (see Materials and Methods for more details).

### Cumulative relative entropy

For each individual genome, CRE values were calculated by increasing the *k*-mer length from 5 to 15. We plotted CRE values for the 3,905 referenced viral genomes (Fig. 2), which are colored by genome size and ordered from smallest to largest genome. CRE curves did not simultaneously drop to <10% of the maximum CRE value for all genomes, which is the selection criterion that Wu *et al*.^32^ recommended. When curves for the smaller genomes achieve that goal, some curves for larger genomes are still at a plateau. At *k* = 9, the curves of small genomes start to fall <10% of the maximum CRE, and ~50% of all CREs drop to <20% of their maxima. At larger values of *k (k* = 10, 11, and 12), more genome CREs satisfy the <10% of the maximum criterion. When *k* = 13, the CRE values of most genomes fall to <10% of the maximum CREs. However, *k* = 13 cannot simply be chosen as the optimal feature length, because it might be too large (no information left) for small genomes. By quartile, the optimal *k*-mer lengths for subgroups Q1, Q2, Q3, and Q4 are determined to be 9 to 11, 10 to 12, 11 to 13, and 12 to 15, respectively. Therefore, we initially determined the optimal range of *k*-mer lengths for the entire set of 3,905 genomes to be 9 to 13. This range will be refined in the following steps.

### Average number of common features

It was previously found that computed RSD values did not work as expected (that is, they did not converge to zero after reaching the optimal feature length). Because of this, we did not use the comparison with random feature space; instead, we only used the denominator of the RSD to explore the average number of common features between pairwise genomes, which we call the ACF. For each genome, the ACF is defined as the average number of common features from a pairwise comparison of all the other genomes at a specific feature length (see Materials and Methods). As FFP is a pairwise-comparison method, the ACF is not expected to be very low among samples at the specific feature length. Otherwise, the obtained information will tend to be randomized, which means that it could produce a random phylogeny. On the other hand, very high ACF will lead to obtain a poor discrimination phylogeny.

**Figure 3:**
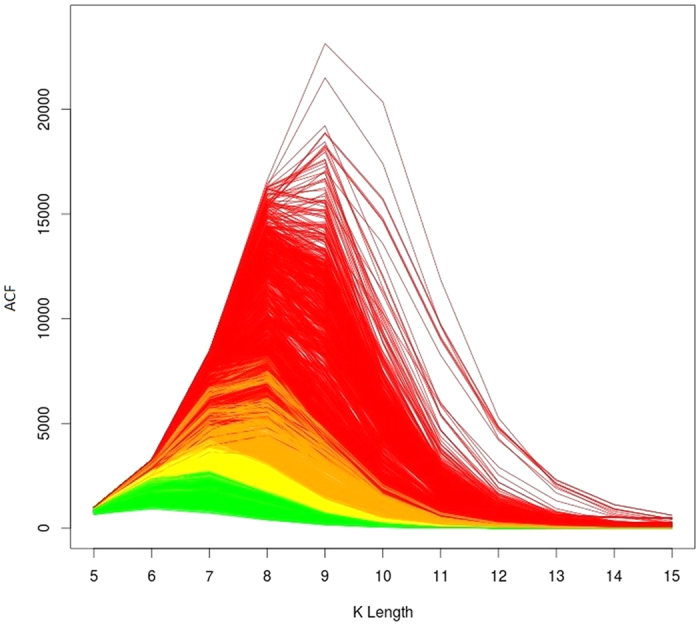
ACF for the 3,905 viral RefSeq genomes. Each curve shows the ACF numbers between this individual genome and the other 3,904 genomes. Subgroups Q1, Q2, Q3, and Q4 are green, yellow, orange, and red, respectively.

First, in order to reveal the shared degree of features at different length, we calculated the ACF among 3,905 RefSeq viral genomes by comparing each genome with the other 3,904 at different feature lengths, as plotted in Fig. 3. The ACF plot demonstrated that few features are shared when the feature length is larger than 11 (*k* > 11). As a result, the maximal feature length for 3,905 genomes should be 11 nucleotides. Therefore, the optimal range for *k* is reduced to 9–11. These curves were also coloured by different genome sizes, as in subgroups Q1–Q4. As seen in the Fig. 3, the ACFs are increased with an increased genome size. As we estimated, when *k* = 13, many of the features of small genomes (Q1 subgroup) are shared, which implies that we cannot consider only the CRE criterion to choose the optimal *k*.

**Figure 4:**
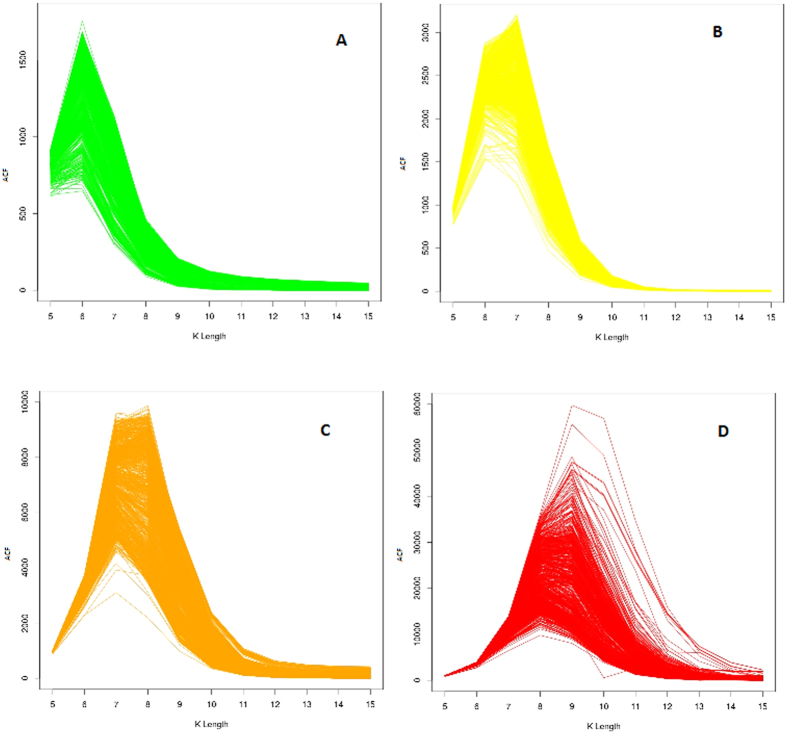
ACF for viral RefSeq genomes in four subgroups. (**A**) Q1 subgroup (genome size <25% quartile): 976 genomes, green; (**B**) Q2 subgroup (genome size in 25–50% quartiles): 977 genomes, yellow; (**C**) Q3 subgroup (genome size in 50–75% quartiles): 977 genomes, orange; (**D**) Q4 subgroup (genome size >75% quartile): 977 genomes, red.

Finally, we also calculated ACF values for the subgroups (Fig. 4), by comparing each genome with the other 995 or 996 in the same quartile. The maximal optimal feature lengths for Q1, Q2, Q3, and Q4 were found to be 10, 11, 12, and 13, respectively. As a result, the optimal feature ranges were reduced to 9–10, 10–11, 11–12, and 12–13.

### All observed feature occurrences in genomes

The unions of all observed features at different lengths have been calculated and compared with theoretical occurrences, as shown in Table 1. Noticeably, the number of observed non-redundant features increases exponentially as powers of alphabetical size (4 for nucleotide sequences); i.e., when *k* < 13, the total redundant feature number (165,838,971) largely covers the expected feature space. However, when *k* > 13, the number of observed non-redundant features grows more slowly. In the subgroups, all of the feature numbers show similar trends.

**Figure 5:**
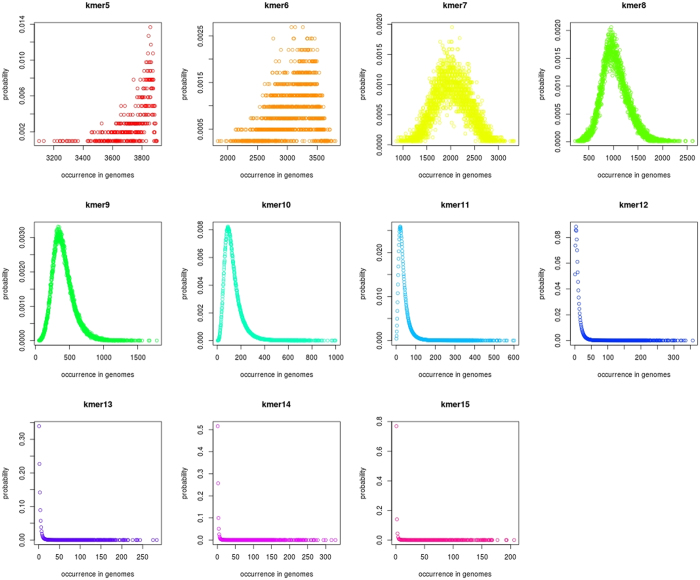
Distribution of feature occurrences in genomes. A dot represents a unique *k*-mer. The *y*-axis represents probability (*k*-mer fraction) calculated from the observed frequency of individual *k*-mers divided by total number of observed *k*-mer; the x-axis represents the number of genomes that share the same *k*-mer.

**Figure 6:**
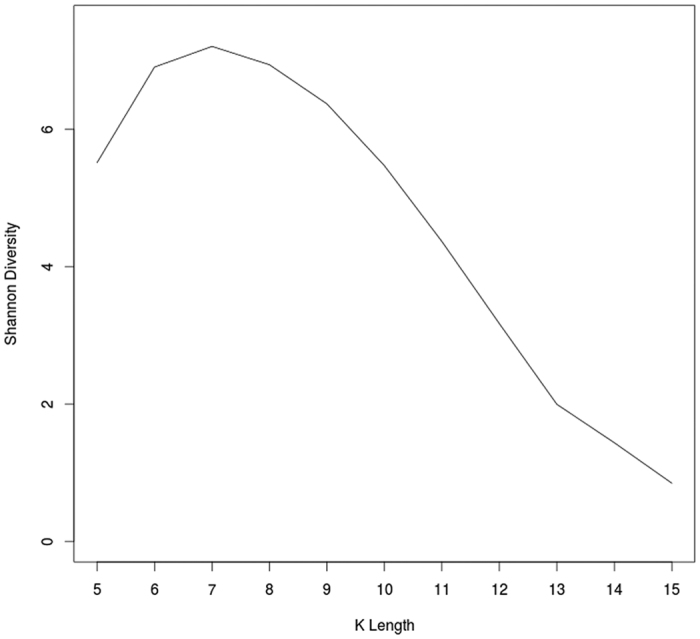
The Shannon diversity index for feature occurrences in genomes as a function of *k*-mer length.

The optimal *k*-mer length necessary to construct a good dendrogram is that length that provides the best balance of shared and unique features among the genome dataset. To illustrate the relationship between “all features” and “all genomes”, the distribution of feature occurrences in genomes was calculated and plotted. As shown in Fig. 5, when the feature length is small (*k* = 5, 6), most features can be found in most genomes; however, when the feature length is large (*k* = 14, 15), most features (>50% or 80%) are unique (occurrence = 1). In either scenario, the FFP method cannot work efficiently. After all, the feature occurrences should be diverse to balance the similarity and dissimilarity when comparing all genomes. For this purpose, the Shannon diversity index was applied and plotted with different feature lengths (Fig. 6). From the curve, the diversity of feature occurrence peaks at *k* = 7, and then drops steadily. In this regard, *k* = 9 is more appropriate than 10 or 11 within our previous optimal feature range.

**Figure 7:**
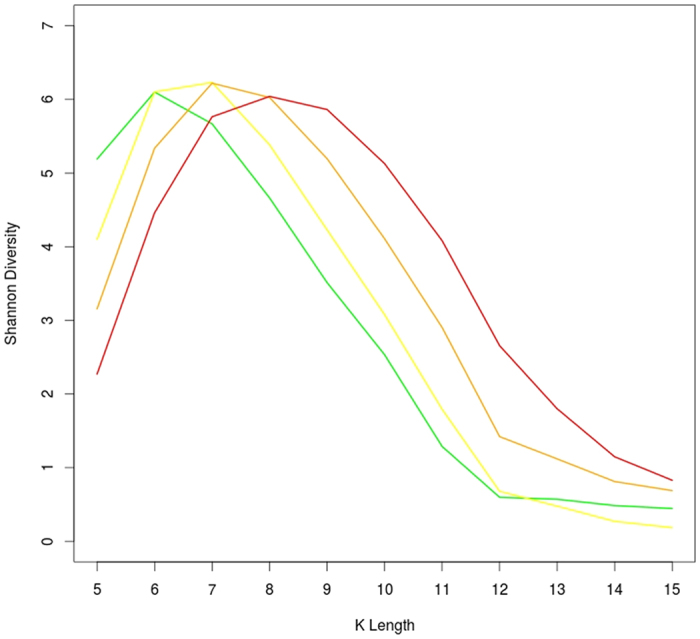
The Shannon diversity index for feature occurrences in four subgroups as a function of *k*-mer length. Q1 subgroup (genome size <25% quartile): 976 genomes, green; Q2 subgroup (genome size in 25–50% quartiles): 977 genomes, yellow; Q3 subgroup (genome size in 50–75% quartiles): 977 genomes, orange; Q4 subgroup (genome size >75% quartile): 977 genomes, red.

We repeated the same process for each of the four subgroups and obtained Supplementary Figures S1–S4 for distributions and Fig. 7 for the Shannon diversity index. Considering all criteria, the optimal feature lengths for Q1, Q2, Q3, and Q4 were determined to bed 9, 10, 11 and 12, respectively.

### What is the optimal feature length?

**Figure 8:**
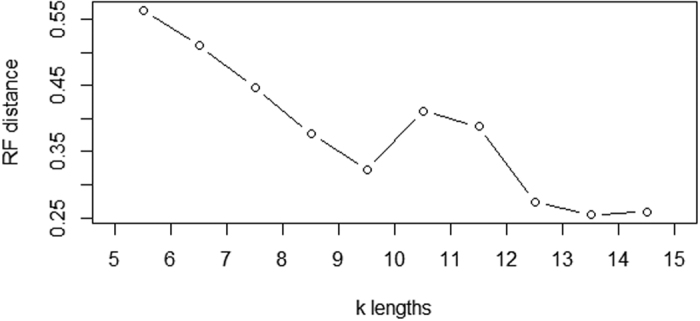
The Robinson–Foulds distance between trees at feature length *k* (5, 6, 7, …) and *k* + 1.

The results for the application of all criteria to the selection of optimal *k*-mer length are summarized in Table 2. For the dendrogram of the 3,905 viral genomes, either 9 or 11 can be chosen as the optimal feature length. *k* = 10 has a lower ACF and Shannon diversity index, indicating a non-linear relationship in the dataset. When *k* = 9, CRE values have not yet dropped to <10% of their maximum, and the other two criteria perform well. When *k* = 11, most of the CRE values have droped to <10% of their maximum, although the ACF is not good for small viral genomes. In this case, it is hard to choose between 9 and 11, because neither can perfectly satisfy our three criteria. Therefore, it makes sense to check the tree stability and use it as supporting information for this study. To evaluate the tree stability, we calculated Robinson–Foulds distances between *k* (5, 6, 7…) and *k* + 1 at different feature lengths. When the Robinson–Foulds distances drop to a low value, it means that the tree stability starts at this *k* point and the tree topology does not change much as feature lengths increase. As shown in Fig. 8, the trees start to converge at *k* = 9; therefore, we will choose *k* = 9 as the optimal feature length for this dendrogram. Furthermore, because we want to obtain a global view of the relationship among RefSeq viral genomes, the “pairwise comparison perspective” and “all genome comparison perspective” are considered more important in this research than the exact estimation of individual genomes, especially when all sequences are RefSeq whole genomes (not as similar and sensitive). For dendrograms of the four subgroups Q1, Q2, Q3, and Q4, the optimal feature lengths have been identified as *k* = 9, 10, 11, and 12, respectively.

### Phylogenomic analysis of 3,905 viral RefSeq genomes

**Figure 9:**
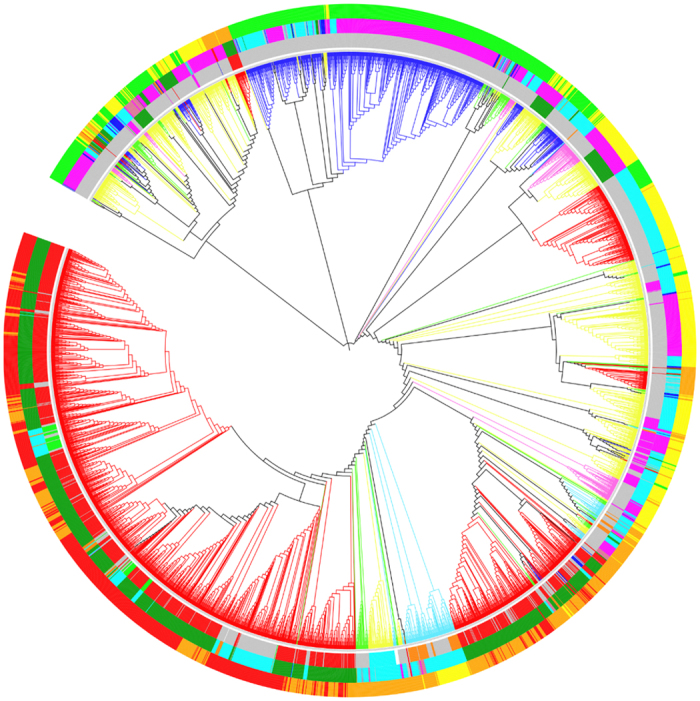
Optimal dendrogram of the 3,905 RefSeq viral genomes (*k* = 9). The braches are colored by Baltimore classifications. The circles, from inside to outside, are colored by different orders, hosts, and genome sizes as follows: The ranches by Baltimore classification: dsDNA (no RNA stage), red; dsRNA, green; RT viruses, pink; ssDNA, blue; ssRNA negative-strand, bright blue; ssRNA positive-strand, yellow. The First circle, inside to outside, by order: *Caudovirales*, red; *Herpesvirales*, green; *Ligamenvirales*, blue; *Mononegavirales*, orange; *Nidovirales*, cyan: *Picornavirales*, pink; *Tymovirales*, dark green; unclassified, silver. The second circle, inside to outside, by host: protist, orange; archaea, red; bacteria, dark green; fungi, blue; animal, cyan; animal and plants, pale violet red; plant, pink; environment or NA, silver.The third circle, inside to outside, by genome size: Q1, green; Q2, yellow; Q3, orange; Q4, red.

Based on the three-step assessment, the dendrogram of all 3,905 RefSeq viruses (*k* = 9) is shown in Fig. 9. This dendrogram was built by the neighbour-joining method using all FFP values as pairwise distances. As a whole, the taxonomic groupings of the 3,905 viral whole genomes generally agree well with the reference taxonomy. The dendrogram is colour-coded by the Baltimore classification, viral orders, kingdoms of hosts, and by genome sizes. From this dendrogram, a global view of all relationships among the 3,905 viral RefSeq genomes is demonstrated. We used this dendrogram as a preliminary step to show the global view of clustering for the hundreds of whole genomes of Ebola viruses sequenced in the 2015 West Africa Outbreak compared with the diverse set of viral taxa. We then used rigorous analysis based on traditional methods to analyse the genomic variation among Ebola viruses^40^.

As shown in Fig. 9, all branches of the dendrogram are coloured by the Baltimore classification, including dsDNA viruses, dsRNA viruses, retrotranscribing (RT) viruses, ssDNA viruses, ssRNA positive-strand viruses, and ssRNA negative-strand viruses. In the dendrogram, dsDNA viruses, the largest taxon, are classified into five major groups, which include one relatively large group, one medium-size group, and three small groups. The second major group, ssRNA(+) viruses, forms multiple small clades and interlaces among other groups. ssDNA viruses also form five groups, which include one large group and four small groups. ssRNA(−) viruses and RT viruses organize two relatively independent clades.

The innermost circle of the dendrogram is coloured by reference taxonomy at different *Orders*, including *Caudovirales, Herpesvirales, Ligamenvirales, Mononegavirales, Nidovirales, Picornavirales, Tymovirales*, and unclassified families. Supplementary Table S1 shows that ~60% of viruses in our dataset are *Caudovirales* (excluding the 2,171 viruses whose reference orders are unclassified or unassigned). In general, the *Caudovirales* viruses group well (ignoring the unclassified orders), with a few member discrepancies. It is interesting to note, however, that *Herpesvirales* viruses form a small clade to split the largest clade of *Caudovirales*. Other *Herpesvirales* viruses also group inside the *Caudovirales* clades as discrepancies. The *Ligamenvirales, Mononegavirales, Nidovirales, Picornavirales*, and *Tymovirales* orders separate from each other to form small sporadic groups.

The second circle shows the hosts’ kingdoms, including archaea, bacteria, fungi, animal, plants, protists, and the environment. As shown, the host kingdom with the most dsDNA viruses is bacteria. The plant viruses are mainly ssDNA viruses and ssRNA(+) viruses. The animal viruses distribute around the whole dendrogram, and respond to various sequence structures and reference orders, which suggests their possible origin from transmission. Different levels of genome sizes color the outside circle. The overall trend is that genomes with similar sizes are easier to group together, although the colours mix as locality changes.

From Fig. 9, we observed that there is a correlation between the length of the genome and dendrogram grouping as shown in the outer circle. Therefore, the dendrogram of subgroup based on the optimal *k*-mer as reported in Table 2, will give a better taxonomic resolution.

### Statistical analysis for grouping uncertainty

The RefSeq dataset of 3,905 genomes contains 97 known families (by the ICTV annotation), and 59 genomes do not have information about their families (missing or “unassigned” in GenBank). The 10 largest families (see Material and Methods) were evaluated for grouping uncertainty (Huang *et al*.^34^). Considering the dendrogram derived from the optimal *k* = 9, the descriptive statistics of within-group and between-group distances of different viral families were calculated by the Kruskal–Wallis one-way ANOVA and the Wilcoxon rank sum test. For the Kruskal–Wallis one-way ANOVA, the null hypothesis, which is that the within-group and between-group distances of the largest 10 families have equal means, is rejected (*p* < 2.2 × 10^−16^). The pairwise Wilcoxon rank sum test shows that within-group distances are smaller than the between-group distance for each viral family (*p* < 2.2 × 10^−16^). These statistical results strongly indicate a good grouping of the constructed dendrogram and its consistency with ICTV annotation. Detailed results of the statistical analysis are provided in Supplementary Table S2.

### Subgroup dendrograms

The dendrogram (*k* = 9) for the 976 RefSeq viral genomes in subgroup Q1 (genome size <25%) is shown in Supplementary Figure S5. ssDNA viruses comprise a majority of this dendrogram and most of them are clustered together to form a large clade (blue branches). This clade is separated by two main viral hosts: plants and animals. The other large clade of animal viruses is formed by two independent clusters of ssDNA and dsDNA. ssRNA(+), dsRNA, and RT viruses can also be observed. These three classes form independent small clades and then cluster with each other, likewise with grouping resulted by the host information. The orders of most viruses in subgroup Q1 are unclassified, except for some *Tymovirales*.

Supplementary Figure S7 shows a dendrogram (*k* = 11) for the 977 RefSeq viral genomes in subgroup Q3 (genome size 50–75%). More than 60% are dsDNA viruses. They are clustered together in this dendrogram, and most of them are in the *Caudovirales* family and bacterial viruses, although some special cases are either archaea viruses in the *Ligamenvirales* family or unclassified animal viruses. The other 40% of viruses in this dendrogram are mainly ssRNA(+), ssRNA(−), and dsRNA. Each classification forms a few small clusters and then group with the others. It is worth noting that animal ssRNA(+) viruses are closer to animal dsRNA viruses than to plant ssRNA(+) viruses, although the latter are in the same classification. Also, in this dendrogram, *Mononegavirales* viruses have an independent clade with different hosts.

For the largest viruses (Q4; genome size >75%).), most of them are dsDNA viruses (Supplementary Figure S8). The *Caudovirales* viruses, most of which are bacterial viruses, form three large clades. Among these are animal viruses with a few protist viruses whose orders are *Herpesvirales* or unknown.

## Discussion

Identifying optimal feature length in an alignment-free phylogenomic method is an important but challenging process, especially when we construct phylogenomic trees for large-scale datasets of divergent genomes of varied size. In this study, we have developed a comprehensive strategy to find the optimal length of *k*-mers in alignment-free phylogenomic analysis, and we built phylogenomic dendrograms for all complete viral genomes in the NCBI RefSeq as of October 2014^41^.

With the development of sequencing technologies, whole-genome information presents new possibilities for microbial classification^42^. Compared to traditional gene trees, whole-genome phylogenies use completed genomic information and solve the incongruence generated by gene trees from various studies. The alignment-free method with *k*- mers is useful for comparing genomes with low homology and has been applied to various microbial studies. However, it is still not clear how to find the optimal feature length of *k*-mers in alignment-free phylogenomic analysis, especially for large-scale comparison of viral genomes. CRE and RSD values have been used as criteria in previous studies^29^,^31^,^32^,^39^, however, these studies used, at most, hundreds of genomes and their lengths did not change greatly. However, thousands of viral genomes in the NCBI RefSeq showed a great difference in size, which ranged from the smallest (*Anguilla anguilla* circovirus) at 1,378 nucleotides to the largest (*Pandoravirus salinus*) at 2,473,870 nucleotides. As a result, their CRE curves cannot simultaneously drop to <10% of maximum as required in previous studies. Furthermore, CRE reflects the ability to identify individual whole genomes at various lengths of *k*. More details should be taken into consideration when dealing with such highly-diverse data, such as pairwise-comparison information and shared *k*-mers among all genomes. Hence, we divided our dataset into four subgroups by 25%, 50%, and 75% quantiles cut-off of genomic size.

In this study, we designed a comprehensive strategy to find the optimal length of *k*-mers for alignment-free FFP phylogenomic analysis. This comprehensive strategy combines three steps: (1) an individual genome perspective: CRE to find the minimum feature length; (2) pairwise-comparison perspective, where the ACF among genomes is applied to determine the maximum feature length; (3) an all-genome comparison perspective, where the Shannon diversity index of all observed feature occurrences in genomes was used to find the optimal feature length between the minimum and the maximum. If the results are not unique, the tree stability information (obtained from the Robinson–Foulds distance) can be used to determine the optimal length of *k*. Based on these criteria, the optimal feature lengths for each subgroup have been identified and are shown in Table 2. To trace the global relationship of all 3,905 viral whole genomes, we chose the smallest *k (k* = 9) among the optimal feature lengths for subgroups as an acceptable feature length and constructed a dendrogram of all viral whole genomes.

In conclusion, our three-step comprehensive strategy was successfully applied to identify the optimal feature length of *k* in an alignment-free phylogenomic analysis for thousands of whole genomes with highly-diverse sizes. Moreover, our dendrogram with the optimal feature length derived from all complete viral genomes provides a global view of classification in good agreement with the current viral taxonomy reported by the ICTV and the Baltimore classification. Moreover, this overall dendrogram can also be used as a preliminary step to show the global view of clustering of the diverse viral taxa and further analyze the genomic variation by traditional methods for specific viruses as example on the study of Ebola viruses that were responsible for the recent outbreak in 2015 in West Africa^40^.

## Materials and Methods

### Dataset

Viral genomes (5,326) were downloaded from the RefSeq database^41^ (http://www.ncbi.nlm.nih.gov/refseq/) in October of 2014. After merging all multiple-segmented genomes from the same virus, 4,300 genomes were obtained. Viroid and satellite data were excluded from the dataset, and then 3,905 genomes remained for this research. All genome data were converted to *k*-mer feature counts with Jellyfish software^43^. The dataset was also divided into four subsets by 25%, 50%, and 75% quantile cut-offs of genomic size in order to explore how optimal feature lengths vary by genomic size.

### FFP and phylogenomic trees

All phylogenomic trees were calculated based on FFP-based distance matrices^29^. All criteria, which are related to optimal feature lengths, were computed in parallel with Python 2.7. Phylogenomic trees were calculated from distance matrices based on the neighbor-joining method with the R package phytools^44^. All dendrograms were plotted by the ITOL online tool (http://itol.embl.de/itol.cgi), and the other figures were generated by R software.

### Optimal feature lengths

**Figure 10:**
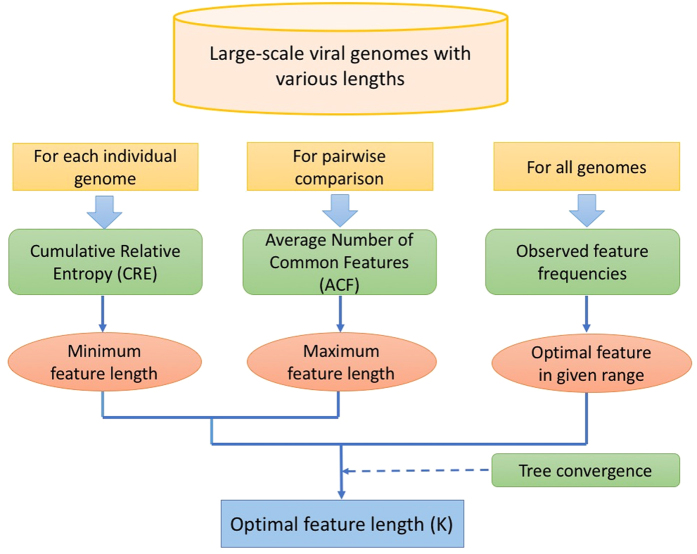
The three steps of assessment to obtain optimal feature lengths (*k*).

As shown in Fig. 10, the optimal feature lengths were determined by three criteria: (1) from the individual genome perspective using CRE; (2) from a pairwise-comparison perspective: ACF among genomes; and (3) from an all-genome-comparison perspective: all observed feature occurrences in genomes. If multiple values of feature lengths were determined after this process, tree stability will be used to find the optimal length.

#### Cumulative relative entropy

A general description of CRE was provided previously^39^, and the optimal feature length, *k,* was considered the point where the genome curves start having zero CRE or begin falling to <10% of their CRE maximum values. The CRE has been calculated as follows^32^:


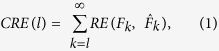


and


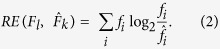


Where *l* is the feature length, *f*_*i*_ is the observed feature frequency, and 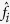 is the expected frequency formulated from the Markov chain of order 2 as in the previous study^45^. Because the relative entropy (Kullback–Leibler divergence)^46^ is always a non-negative value, the function of CRE is monotonically decreasing.

In previous studies^29^,^32^, RSD has also been used to determine the optimal feature length. However, RSD cannot be applied for this research because our 3,905 genomes provide a huge feature space, and the overlap in feature space between the viral genomes and random sequence does not reduce. As a result, not all RSD values decrease to zero as expected. From another aspect, the random sequences are only generated once, without any iteration, and the iteration can be time-consuming. Therefore, RSD was discarded for use e in this research. However, from this information, we developed the ACF to check the overlap in feature space among genomes.

#### Average number of common features

For pairwise genomes, the similarity in FFP methods is actually held by the common features between them. When *k* is small, most features in one viral genome can be shared by another. However, the all possible features number is small (4 ^*K*^), therefore the average number should be low. On the other hand, when *k* is very large (because the features are long), only a few features can be shared between pairwise genomes. In this case, FFP may not provide enough signals for phylogeny and may show a random phylogeny. Therefore, the optimal *k* should be chosen before the ACF drops to low values. The ACF can be defined as follows:


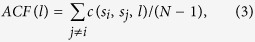


where *c(s*_*i*_, *s*_*j*_, *l*) is the number of common features of length *l* between sequences *s*_*i*_ and *s*_*j*_, and *N* is the genomic number of genomes in the dataset. We used 10% of the maximum ACF for the considered population as the suggested cutoff, similar to the suggestion for RSD^29^,^32^.

#### All observed feature occurrences in genomes

From the perspective of all genomes, to balance the similarity and dissimilarity, neither of these situations are acceptable for FFP: (1) most features can be found in most genomes (when the feature length is too small); and (2) most features are unique (when the feature length is too large). For this purpose, the union of all observed features at different *k* were calculated in our dataset, as well as their occurrence in genomes. Theoretically, the maximum number of all possible features is 4^*k*^. However, the biological sequences are not a random combinations of alphabets (nucleotide bases). As a result, the percentage of observed features relative to the maximum possible number of features decreases as feature length increases in our 3,905 genomes. To balance the level of similarity and dissimilarity, the occurrence for all observed features can be measured by the Shannon diversity index^47^:


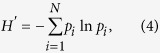


where *p*_*i*_ is the probability that features can be found in *i* genomes and *N* is the total number of genomes in the dataset. For the specific length *k*, the number of observed *k*-mers is *O*_*k*_ (*O*_*k*_ ≤ 4^k^). *C*_*i*_ *k*-mers, can be found in *i* genomes (1 ≤ *i* ≤ *N*). The *p*_i_ can be calculated as *p*_*i*_ = *C*_*i*_/*O*_*k*_. For example, to calculate the Shannon diversity index of the *k* = 9 dendrogram, the *O*_*k*_ = 262,144. We assume there are *C*_*5*_ is the number of *k*-mers that can be found in five genomes, which means that *C*_*5*_ many *k*-mers exist in five of the 3,905 genomes. Here *p*_*5*_ = *C*_*5*_/262,144 (*i* = 5). The Shannon diversity index can be calculated by adding values from *p*_*1*_ to *p*_*3905*_.

#### Tree stability

Although the three-step process is applied to check the optimal feature length, it is still possible that inconsistent results can be obtained from the three criteria. To strengthen the feasibility of our method, we used tree stability as an additional factor to determine the optimal feature length. Tree stability is estimated by calculating the topology difference between trees at feature length *k (k* = 5, 6, 7, ……) and *k* + 1 using Robinson–Foulds distance^48^, which is a metric to compare differences between two phylogenies. Therefore, when the Robinson–Foulds distances between trees at feature length *k* and *k* + 1 decrease to a low value, it means that the tree stability starts at this *k* point and the tree topology does not change much as *k* increases. In our case, trees start to converge at *k* = 9; therefore, *k* = 9 was chosen as the optimal feature length for the global dendrogram.

### Evaluation of grouping uncertainty

The dendrogram (*k* = 9) was evaluated for grouping uncertainty by viral family annotation, based on ICTV classification, using the statistical methods described by Huang^34^. Kruskal–Wallis one-way ANOVA was used to evaluate the difference of the distance mean between within-groups and between-groups. The Wilcoxon rank sum test was used to evaluate the difference of distance means between within-group and between-group for each group. The top 10 highest members of viral families, which are^1^ S*iphoviridae* (657 viruses), *Geminiviridae* (364 viruses), *Myoviridae* (307 viruses), *Podoviridae* (218 viruses), *Papillomaviridae* (125 viruses), *Potyviridae* (119 viruses), *Parvoviridae* (81 viruses), *Picornaviridae* (73 viruses), *Flaviviridae* (70 viruses), and *Betaflexiviridae* (66 viruses) were selected to perform the statistical analyses.

## Additional Information

**How to cite this article**: Zhang, Q. *et al*. Viral Phylogenomics Using an Alignment-Free Method: A Three-Step Approach to Determine Optimal Length of *k-mer.* *Sci. Rep.* **7**, 40712; doi: 10.1038/srep40712 (2017).

**Publisher's note:** Springer Nature remains neutral with regard to jurisdictional claims in published maps and institutional affiliations.

## Supplementary Material

Supplementary Information

## Figures and Tables

**Table 1:** Numbers of all observed non-redundant features in the 3,905 genomes and in subgroups.

*k*	Expected (4^k^)	Observed	Observed in subgroups			
			Q1	Q2	Q3	Q4
5	1,024	1,024	1,024	1,024	1,024	1,024
	%obs/exp	100	100	100	100	100
6	4,096	4,096	4,096	4,096	4,096	4,096
	%obs/exp	100	100	100	100	100
7	16,384	16,384	16,384	16,384	16,384	16,384
	%obs/exp	100	100	100	100	100
8	65,536	65,536	65,536	65,536	65,536	65,536
	%obs/exp	100	100	100	100	100
9	262,144	262,144	261,744	262,135	262,144	262,144
	%obs/exp	100	99.84	99.99	100	100
10	1,048,576	1,048,576	927,225	1,028,114	1,048,272	1,048,576
	%obs/exp	100	88.42	98.04	99.97	100
11	4,193,940	4,193,940	1,983,092	3,133,972	4,011,469	4,191,555
	%obs/exp	99.99	47.28	74.72	95.64	99.94
12	16,777,216	16,405,985	2,691,077	5,776,434	10,767,534	15,878,890
	%obs/exp	97.79	16.04	34.43	64.17	94.64
13	67,108,864	48,841,160	2,999,146	7,352,145	17,313,110	41,880,927
	%obs/exp	72.78	4.46	10.95	25.79	62.40
14	268,435,456	87,268,900	3,134,521	7,979,080	20,718,374	67,931,028
	%obs/exp	32.51	1.16	2.97	7.71	25.30
15	1,073,741,824	111,123,028	3,211,835	8,210,153	22,064,213	83,014,712
	%obs/exp	10.35	0.29	0.76	2.05	7.73

The total number of redundant features for 3,905 genomes is 165,838,971. All percentages are calculated based on expected value %obs/exp = percent of observed/expected *k*-mers.

**Table 2:** Summary for optimal feature length.

	Whole dataset	Q1	Q2	Q3	Q4
Step 1: CRE	9, 10, 11, 12, 13	9, 10, 11	10, 11, 12	11, 12, 13	12, 13, 14
Step 2: ACF	9, 10, 11	9, 10	10, 11	11, 12	12, 13
Step 3: featureOccurrence in genomes	9 or 11*	9	10	11	12
Optimal feature length	9 or 11*	9	10	11	12

*k = 9 performs best in Step 3 and *k* = 11 performs best in Step 1.
