# Supplementary material for: Viral Phylogenomics Using an Alignment-Free Method: A Three-Step Approach to Determine Optimal Length of k-mer
Source: Sci Rep. 2017 Jan 19;7:40712. doi: 10.1038/srep40712 (PMC5244389; doi:10.1038/srep40712)
Supplement: Supplementary Information [file srep40712-s1.pdf]

Supplementary information

Viral Phylogenomics Using an Alignment-Free Method: A Three-Step Approach to Determine Optimal Length of *k-mer*

Qian Zhang[2,3], Se-Ran Jun[1,3], Michael Leuze[4,5], David Ussery[1,3], Intawat Nookaew[1,3,*]

[1]Department of Biomedical Informatics, College of Medicine, University of Arkansas for Medical Sciences, Little Rock, AR 72205, USA

[2] UT-ORNL Graduate School of Genome Science and Technology, University of Tennessee, Knoxville, TN 37996, USA

[3]Comparative Genomics Group, Biosciences Division, Oak Ridge National Laboratory Oak Ridge, TN 37831 USA

[4]Joint Institute for Computational Sciences, University of Tennessee, Knoxville, TN 37831, USA

[5]Computational Biomolecular Modeling and Bioinformatics Group, Computer Science and Mathematics Division, Oak Ridge National Laboratories, Oak Ridge, TN 37831, USA

*INookaew@uams.edu

Table S1 Baltimore classification and ICTV Orders Information

Baltimore Classification	counts	ICTV Order	counts
dsDNA viruses, no RNA stage	1826	*Caudovirales*	1208
(+)ssRNA viruses	911	*Picornavirales*	157
ssDNA viruses	649	*Tymovirales*	141
dsRNA viruses	192	*Mononegavirales*	91
(-)ssRNA viruses	180	*Herpesvirales*	67
Retro-transcribing viruses	131	*Nidovirales*	58
Unclassified viruses	8	*Ligamenvirales*	12
Unclassified virophages	5	Unassigned or Unclassified	2171
Unassigned ssRNA viruses	3		

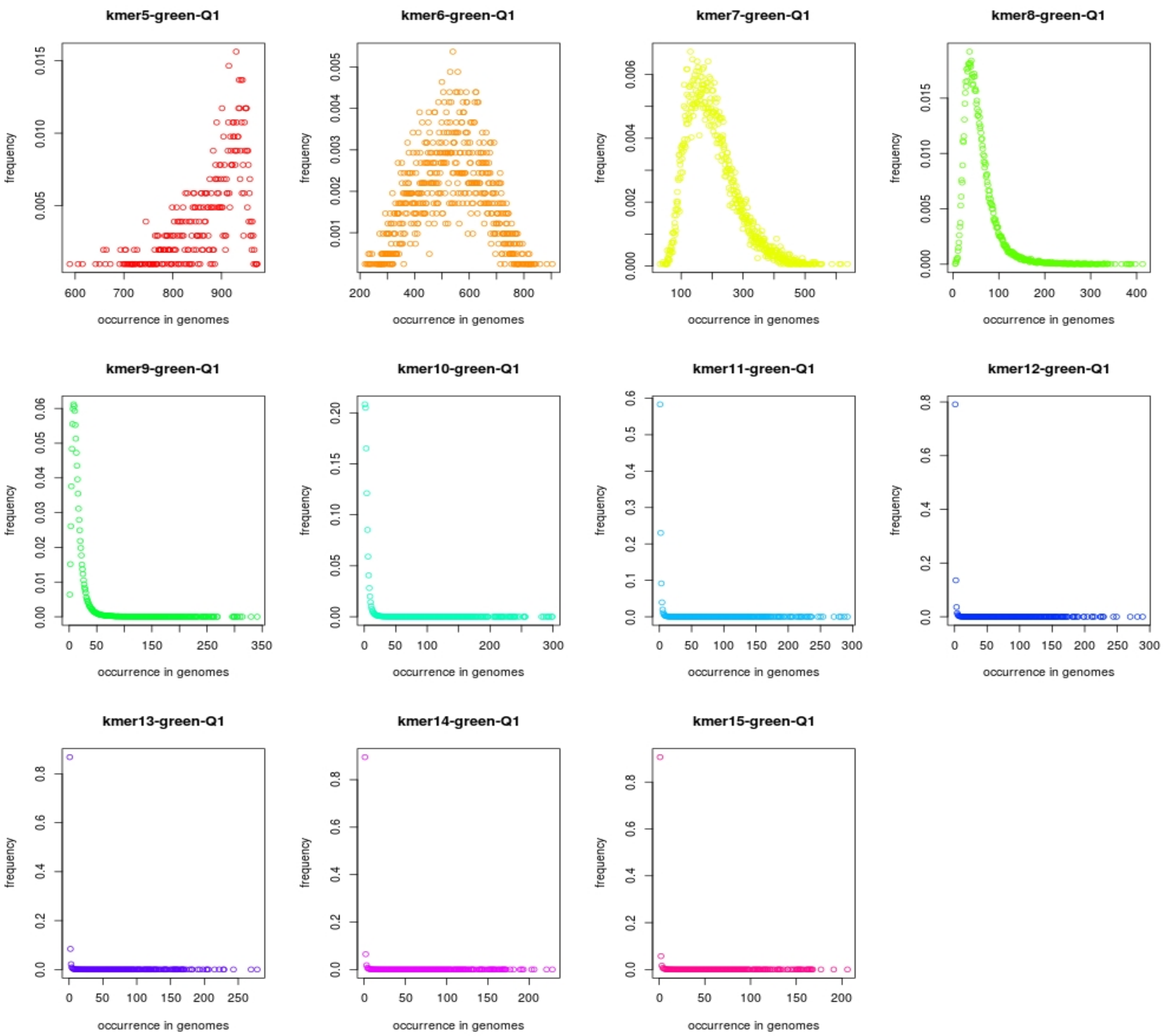

Figure S1 Distribution of feature occurrences in subgroup Q1 (size < 25%)

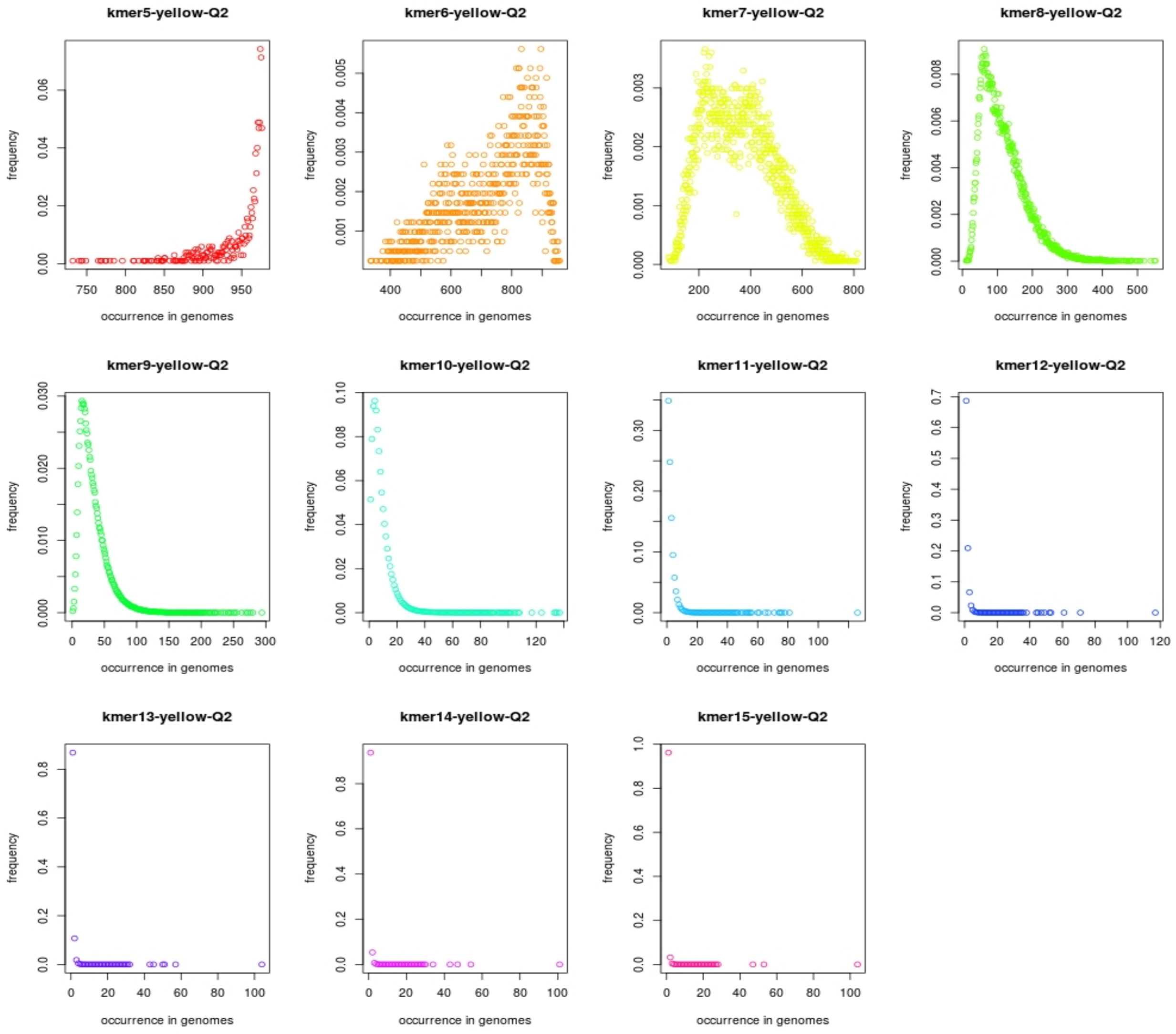

Figure S2 Distribution of feature occurrences in subgroup Q2 (25% < size < 50%)

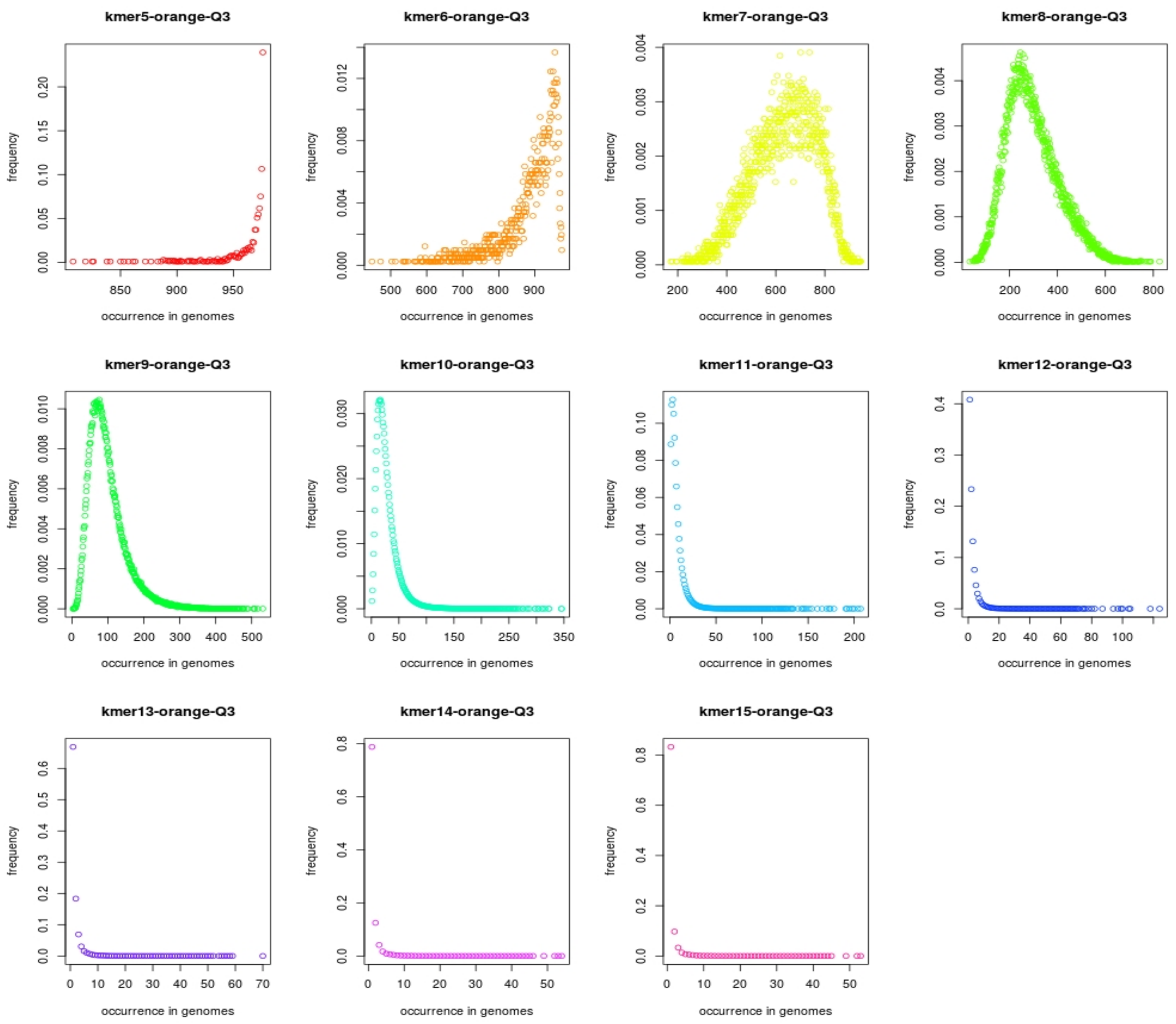

Figure S3 Distribution of feature occurrences in subgroup Q3 (50% < size < 75%)

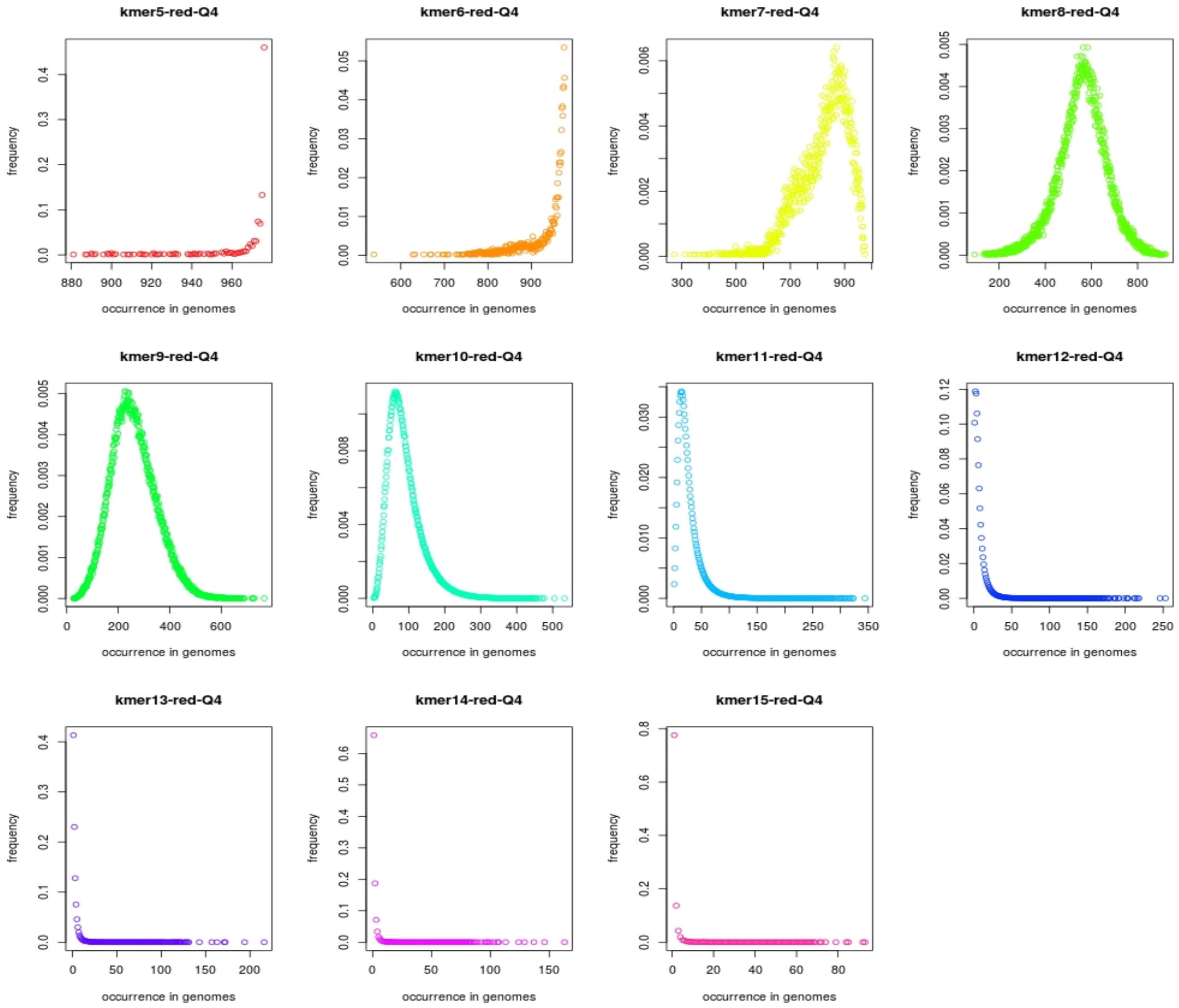

Figure S4 Distribution of feature occurrences in subgroup Q4 (size > 75%)

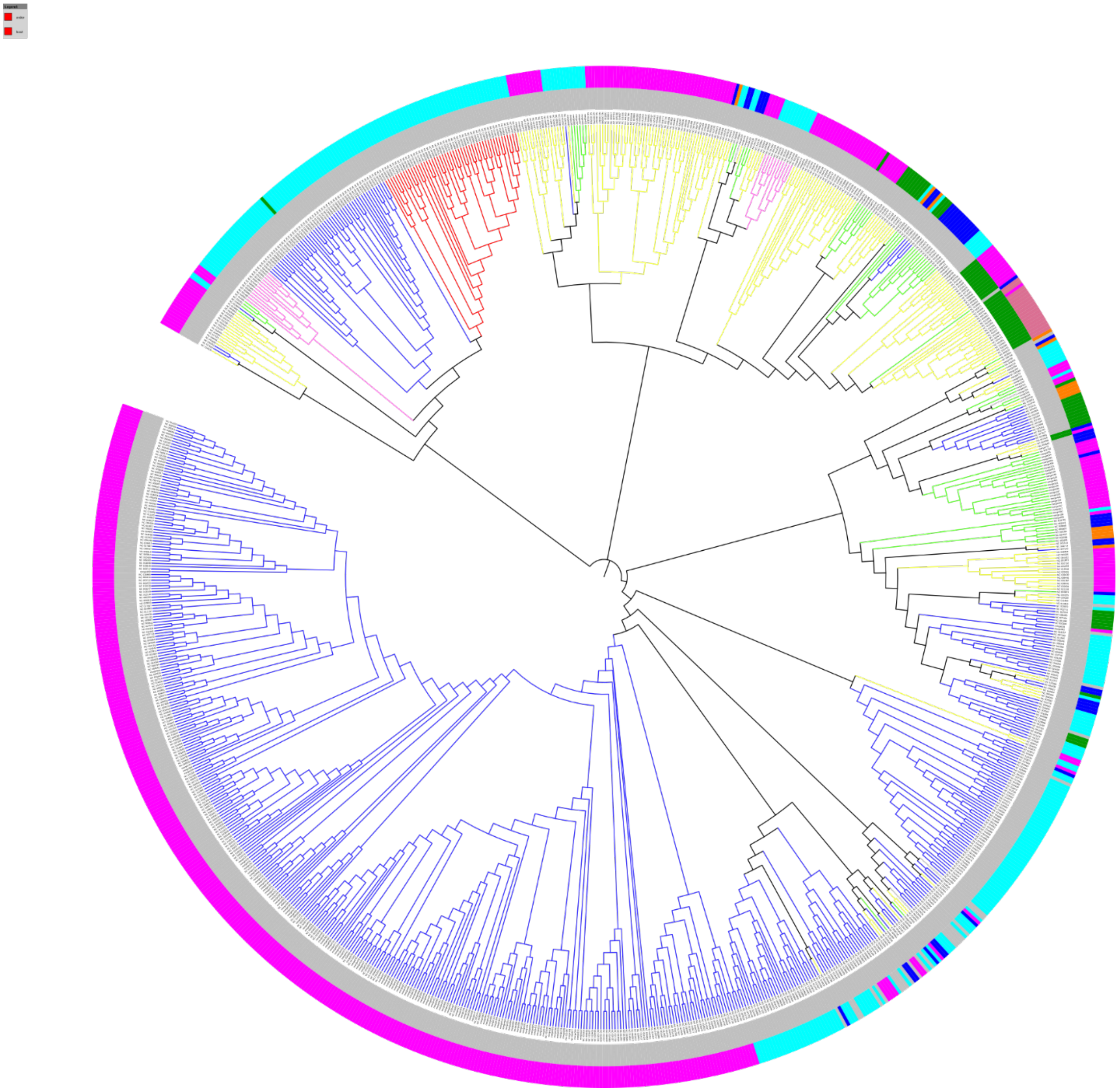

Figure S5 Dendrogram of 976 RefSeq viral genomes in subgroup Q1 (genome size < 25%), when k=9. The braches are colored by Baltimore Classifications. The circles, from inside to outside, are colored by different orders and hosts. [Color information: (A) Branch: Baltimore Classification; dsDNA, no RNA stage: red; dsRNA: green; Retro-transcribing viruses: pink; ssDNA: blue; ssRNA negative-strand: bright blue; ssRNA positive-strand: yellow. (B) From inside to outside, first circle: Order; Caudovirales: red; Herpesvirales: green; Ligamenvirales: blue; Mononegavirales: orange; Nidovirales: cyan: Picornavirales: pink; Tymovirales: dark green; unclassified: silver; (C) From inside to outside, second circle: Host; protest: orange; archea: red; bacteria: dark green; fungi: blue; animal: cyan; animal and plants: pale violet red; plant: pink; environment or NA: silver.]

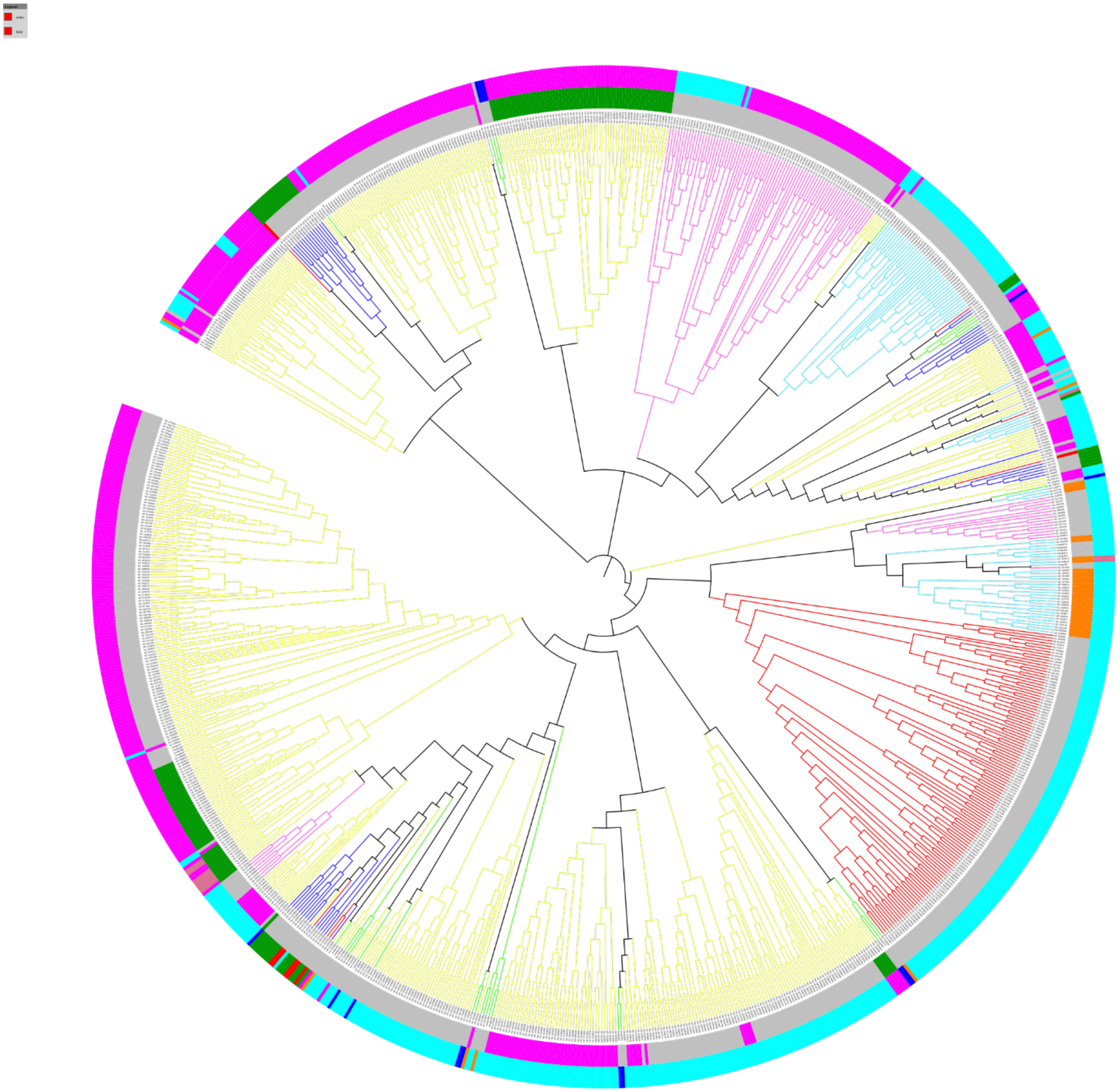

Figure S6 Dendrogram of 977 RefSeq viral genomes in subgroup Q2 (genome size: 25%-50%), when k=10. The braches are colored by Baltimore Classifications. The circles, from inside to outside, are colored by different orders and hosts. [Color information: (A) Branch: Baltimore Classification; dsDNA, no RNA stage: red; dsRNA: green; Retro-transcribing viruses: pink; ssDNA: blue; ssRNA negative-strand: bright blue; ssRNA positive-strand: yellow. (B) From inside to outside, first circle: Order; Caudovirales: red; Herpesvirales: green; Ligamenvirales: blue; Mononegavirales: orange; Nidovirales: cyan: Picornavirales: pink; Tymovirales: dark green; unclassified: silver; (C) From inside to outside, second circle: Host; protest: orange; archea: red; bacteria: dark green; fungi: blue; animal: cyan; animal and plants: pale violet red; plant: pink; environment or NA: silver.]

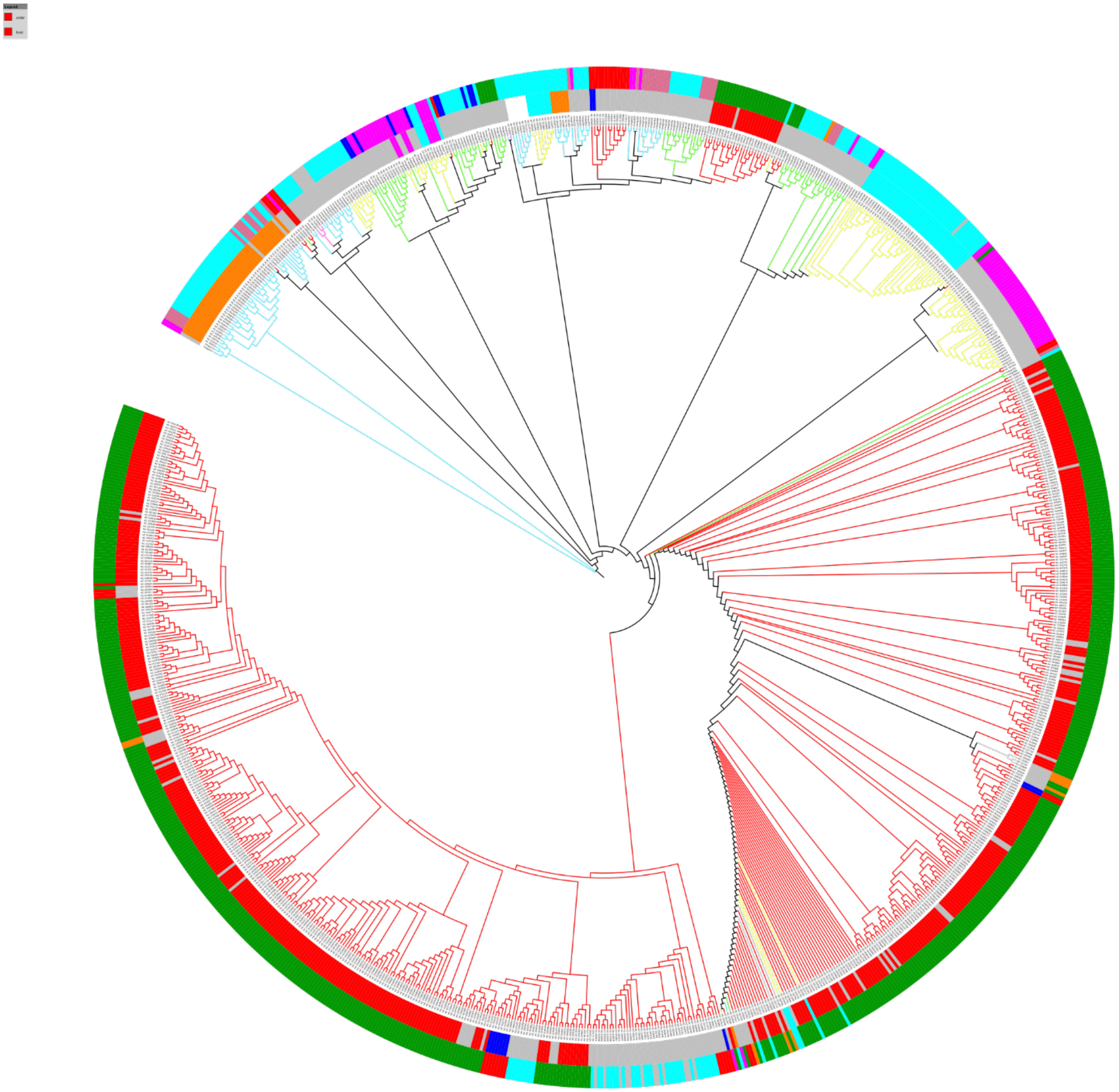

Figure S7 Dendrogram of 977 RefSeq viral genomes in subgroup Q3 (genome size: 50%-75%), when k=11. The braches are colored by Baltimore Classifications. The circles, from inside to outside, are colored by different orders and hosts. [Color information: (A) Branch: Baltimore Classification; dsDNA, no RNA stage: red; dsRNA: green; Retro-transcribing viruses: pink; ssDNA: blue; ssRNA negative-strand: bright blue; ssRNA positive-strand: yellow. (B) From inside to outside, first circle: Order; Caudovirales: red; Herpesvirales: green; Ligamenvirales: blue; Mononegavirales: orange; Nidovirales: cyan: Picornavirales: pink; Tymovirales: dark green; unclassified: silver; (C) From inside to outside, second circle: Host; protest: orange; archea: red; bacteria: dark green; fungi: blue; animal: cyan; animal and plants: pale violet red; plant: pink; environment or NA: silver.]

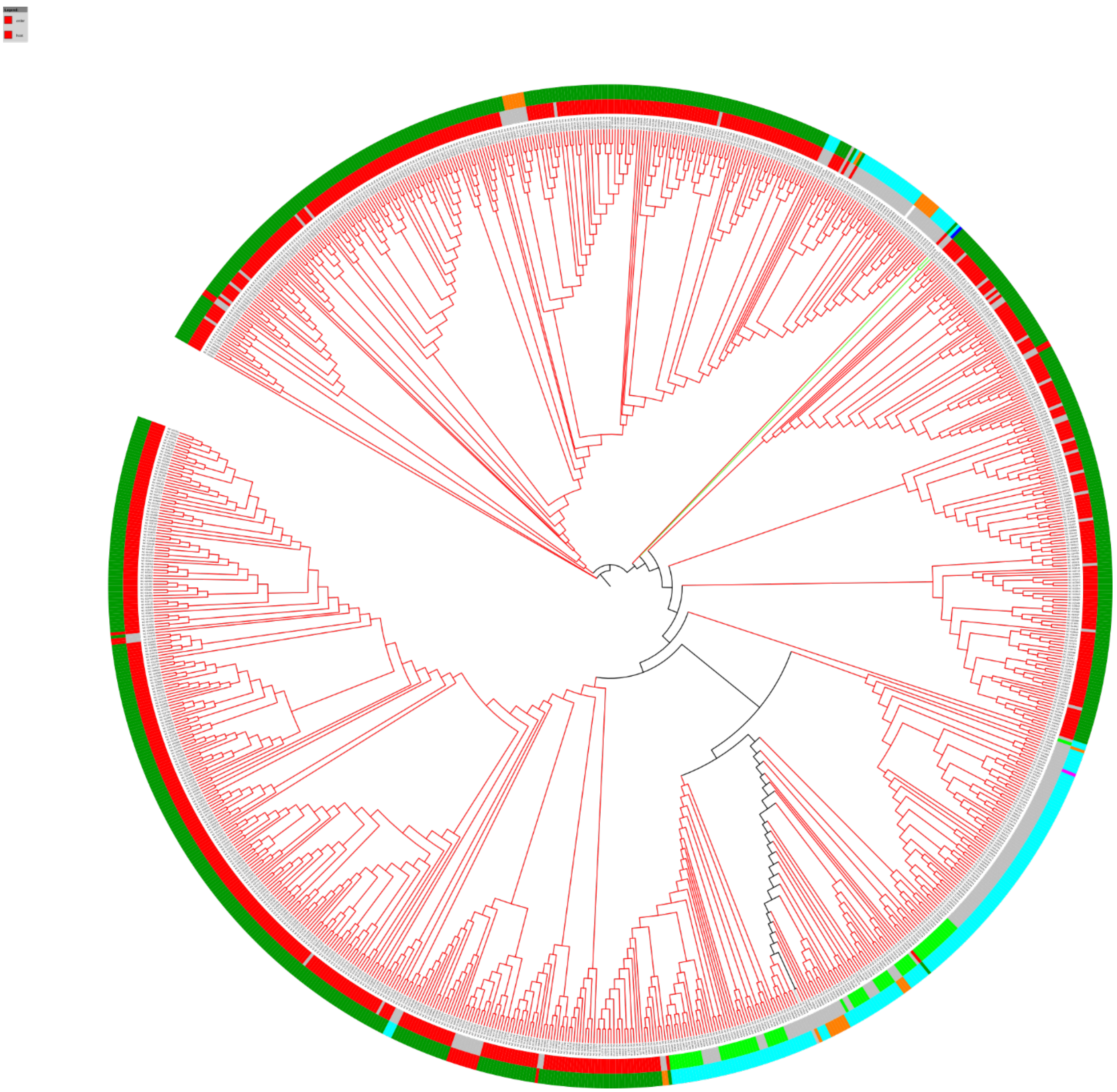

Figure S8 Dendrogram of 977 RefSeq viral genomes in subgroup Q4 (genome size: >75%), when k=12. The braches are colored by Baltimore Classifications. The circles, from inside to outside, are colored by different orders and hosts. [Color information: (A) Branch: Baltimore Classification; dsDNA, no RNA stage: red; dsRNA: green; Retro-transcribing viruses: pink; ssDNA: blue; ssRNA negative-strand: bright blue; ssRNA positive-strand: yellow. (B) From inside to outside, first circle: Order; Caudovirales: red; Herpesvirales: green; Ligamenvirales: blue; Mononegavirales: orange; Nidovirales: cyan: Picornavirales: pink; Tymovirales: dark green; unclassified: silver; (C) From inside to outside, second circle: Host; protest: orange; archea: red; bacteria: dark green; fungi: blue; animal: cyan; animal and plants: pale violet red; plant: pink; environment or NA: silver.]

Table S2 Wilcoxon rank sum test result of the top 10 highest members of viral family

	Siphoviridae	Geminiviridae	Myoviridae	Podoviridae	Papillomaviridae	Potyviridae	Parvoviridae	Picornaviridae	Flaviviridae	Betaflexiviridae
Siphoviridae vs. Geminiviridae	< 2.2 E-16	< 2.2 E-16								
Siphoviridae vs. Myoviridae	< 2.2 E-16		< 2.2 E-16							
Siphoviridae vs. Podoviridae	< 2.2 E-16			< 2.2 E-16						
Siphoviridae vs. Papillomaviridae	< 2.2 E-16				< 2.2 E-16					
Siphoviridae vs. Potyviridae	< 2.2 E-16					< 2.2 E-16				
Siphoviridae vs. Parvoviridae	< 2.2 E-16						< 2.2 E-16			
Siphoviridae vs. Picornaviridae	< 2.2 E-16							< 2.2 E-16		
Siphoviridae vs. Flaviviridae	< 2.2 E-16								< 2.2 E-16	
Siphoviridae vs. Betaflexiviridae	< 2.2 E-16									< 2.2 E-16
Geminiviridae vs. Myoviridae		< 2.2 E-16	< 2.2 E-16							
Geminiviridae vs. Podoviridae		< 2.2 E-16		< 2.2 E-16						
Geminiviridae vs. Papillomaviridae		< 2.2 E-16			< 2.2 E-16					
Geminiviridae vs. Potyviridae		< 2.2 E-16				< 2.2 E-16				
Geminiviridae vs. Parvoviridae		< 2.2 E-16					< 2.2 E-16			
Geminiviridae vs. Picornaviridae		< 2.2 E-16						< 2.2 E-16		
Geminiviridae vs. Flaviviridae		< 2.2 E-16							< 2.2 E-16	
Geminiviridae vs. Betaflexiviridae		< 2.2 E-16								< 2.2 E-16
Myoviridae vs. Podoviridae			< 2.2 E-16	< 2.2 E-16						
Myoviridae vs. Papillomaviridae			< 2.2 E-16		< 2.2 E-16					
Myoviridae vs. Potyviridae			< 2.2 E-16			< 2.2 E-16				
Myoviridae vs. Parvoviridae			< 2.2 E-16				< 2.2 E-16			
Myoviridae vs. Picornaviridae			< 2.2 E-16					< 2.2 E-16		
Myoviridae vs. Flaviviridae			< 2.2 E-16						< 2.2 E-16	
Myoviridae vs. Betaflexiviridae			< 2.2 E-16							< 2.2 E-16
Podoviridae vs. Papillomaviridae				< 2.2 E-16	< 2.2 E-16					
Podoviridae vs. Potyviridae				< 2.2 E-16		< 2.2 E-16				
Podoviridae vs. Parvoviridae				< 2.2 E-16			< 2.2 E-16			
Podoviridae vs. Picornaviridae				< 2.2 E-16				< 2.2 E-16		
Podoviridae vs. Flaviviridae				< 2.2 E-16					< 2.2 E-16	
Podoviridae vs. Betaflexiviridae				< 2.2 E-16						< 2.2 E-16
Papillomaviridae vs. Potyviridae					< 2.2 E-16	< 2.2 E-16				
Papillomaviridae vs. Parvoviridae					< 2.2 E-16		< 2.2 E-16			
Papillomaviridae vs. Picornaviridae					< 2.2 E-16			< 2.2 E-16		
Papillomaviridae vs. Flaviviridae					< 2.2 E-16				< 2.2 E-16	
Papillomaviridae vs. Betaflexiviridae					< 2.2 E-16					< 2.2 E-16
Potyviridae vs. Parvoviridae						< 2.2 E-16	< 2.2 E-16			
Potyviridae vs. Picornaviridae						< 2.2 E-16		0.249381472		
Potyviridae vs. Flaviviridae						< 2.2 E-16			< 2.2 E-16	
Potyviridae vs. Betaflexiviridae						< 2.2 E-16				< 2.2 E-16
Parvoviridae vs. Picornaviridae							0.40400024	< 2.2 E-16		
Parvoviridae vs. Flaviviridae							< 2.2 E-16		< 2.2 E-16	
Parvoviridae vs. Betaflexiviridae							9.69E-14			< 2.2 E-16
Picornaviridae vs. Flaviviridae								0.017555005	< 2.2 E-16	
Picornaviridae vs. Betaflexiviridae								< 2.2 E-16		< 2.2 E-16
Flaviviridae vs. Betaflexiviridae									< 2.2 E-16	< 2.2 E-16
